# Markers of intestinal permeability are already altered in early stages of non-alcoholic fatty liver disease: Studies in children

**DOI:** 10.1371/journal.pone.0183282

**Published:** 2017-09-07

**Authors:** Anika Nier, Anna Janina Engstler, Ina Barbara Maier, Ina Bergheim

**Affiliations:** 1 Department of Nutritional Sciences, Molecular Nutritional Science, University of Vienna, Vienna, Austria; 2 Institute of Nutrition, SD Model Systems of Molecular Nutrition, Friedrich-Schiller University Jena, Jena, Germany; 3 Department of Nutritional Medicine, (180), University of Hohenheim, Stuttgart, Germany; Medizinische Fakultat der RWTH Aachen, GERMANY

## Abstract

**Background & aims:**

Recent studies have shown that patients with manifest non-alcoholic fatty liver disease (NAFLD), e.g. steatosis grade 3 or steatohepatitis with or without beginning fibrosis frequently show altered fecal microbiota composition and elevated bacterial endotoxin levels. However, if these alterations are signs of a progressing disease or are already found in initial disease stages has not yet been clarified.

**Methods:**

Twenty children with simple steatosis (grade 1) diagnosed by ultrasound and 29 normal weight healthy control children (age <10 years) were included in the study (mean age 7.6 ± 1.1 years). Metabolic parameters, markers of intestinal barrier function and inflammation were determined.

**Results:**

Activity of alanine aminotransferase, concentrations of some markers of inflammation and insulin resistance were significantly higher in plasma of NAFLD children than in controls. When compared to controls, plasma bacterial endotoxin and lipopolysaccharide-binding protein (LBP) levels were significantly higher in NAFLD children (+50% and +24%, respectively), while soluble CD14 serum and D-lactate plasma levels as well as the prevalence of small intestinal bacterial overgrowth did not differ between groups. Plasma endotoxin and LBP levels were positive associated with proinflammatory markers like plasminogen activator inhibitor-1, c-reactive protein, interleukin-6 and leptin while no associations with markers of insulin resistance were found.

**Conclusions:**

Taken together, our results indicate that even in juvenile patients with early stages of NAFLD e.g. simple steatosis grade 1, plasma endotoxin concentrations are already elevated further suggesting that intestinal barrier dysfunction might be present already in the initial phases of the disease.

## Introduction

By now, non-alcoholic fatty liver disease (NAFLD), is one of the most frequently diagnosed liver diseases worldwide, not only in adults but also in children and adolescents [1, 2]. Indeed, results of a recent meta-analysis indicate a prevalence of NAFLD of ~8% in children in general population [3]. Furthermore, results of the follow-up study of Feldstein *et al*. suggest that children with NAFLD have a higher risk to develop end-stage liver diseases and a higher mortality rate [4]. However, despite intense research efforts molecular mechanism involved have not yet been clarified and accepted interventions to prevent or cure NAFLD in children, adolescents and adults other than lifestyle modifications are not available [5].

Results of studies in adults with NAFLD suggested already almost 30 years ago that alterations of intestinal microbiota and permeability might be critical in the later stages of NAFLD e.g. fibrosis and cirrhosis [6, 7]. In recent years, it has been shown that similar alterations e.g. increased bacterial endotoxin levels in peripheral blood and small intestinal bacterial overgrowth (SIBO) but also changes of fecal microbiota are also found in patients with manifest steatosis and steatohepatitis with and without beginning fibrosis be it adults or adolescents and even in children [8–13]. In support of the hypothesis that alterations of intestinal microbiota composition and barrier function may be critical in the development and progression of NAFLD, results of several animal studies using different diets to induce NAFLD have shown that interventions affecting intestinal microbiota composition and barrier function can prevent the development of NAFLD [14–21]. However, so far most studies in humans have focused on patients with manifest NAFLD, e.g. steatosis grade 2–3, steatohepatitis or steatohepatitis with beginning fibrosis or even later stages of the disease. Furthermore, in most of these studies cofactors shown to affect the development of NAFLD like smoking, alcohol ingestion but also hormonal changes were present [22, 23], all also discussed to be able to interfere with intestinal microbiota composition and/ or barrier function [24–26]. Indeed, it has not yet been clarified, if an increased translocation of bacterial endotoxin is an independent risk factor causally involved in the onset of NAFLD or if it is only a cofactor in the progression of the disease to later stages. To further delineate if an increased translocation of bacterial endotoxin may add to the onset of NAFLD, we assessed markers of intestinal permeability in prepubertal children (age <10 years) showing early signs of NAFLD, e.g. simple steatosis grade 1 or controls in the present study. Furthermore, it was determined, if these alterations are already associated with metabolic abnormalities and a proinflammatory status.

## Patients and methods

### Human subjects

All children were recruited from the so-called “Hohenheim Fructose Intervention (HoFI) study” and conducted according to the principles of the Declaration of Helsinki of 1975 as revised in 1983. The study was approved by the local responsible ethics committee “Landesärztekammer Baden-Württemberg” (Stuttgart, Germany). All participants and their respective guardians gave written informed consent before the study.

Characteristics of study subjects as well as inclusion and exclusion criteria have previously been detailed by Engstler *et al*. and Maier *et al*. [27, 28]. In brief, for the current analysis, the following inclusion criteria were applied: control group: normal weight (defined as body mass index (BMI) < 90^th^ percentile using reference data for German children [29]); no metabolic abnormalities as defined by Weiß *et al*. [30]; age 5–8 years, NAFLD group: hepatic steatosis; age 5–8 years. None of the children enrolled in the study took drugs or other substances. Age and ethnicity were assessed by questionnaires.

### Liver status and blood parameters

Liver status of all children was assessed by ultrasound by an experienced pediatrician as detailed before by Maier *et al*. [28] using following grading system to assess liver steatosis: grade 0: no steatosis; grade 1: mild steatosis; grade 2: moderate steatosis; and grade 3: severe steatosis [31]. In addition, liver transaminase activity (alanine aminotransferase (ALT) and aspartate aminotransferase (AST)) in serum to determine liver status as well as serum triglycerides, total cholesterol, HDL and LDL cholesterol and fasting glucose concentrations were analyzed by a routine laboratory (Sindelfingen, Germany).

### Anthropometric data, blood pressure and markers of insulin resistance

Body weight, height, waist circumference, blood pressure and homeostatic model assessment for insulin resistance (HOMA-IR) were measured as detailed by Maier *et al*. [28]. Age and gender specific BMI percentiles were assessed using reference data for German children to categorize normal weight and overweight in children [29]. According to the German association of obesity in childhood and adolescence (AGA), BMI standard deviation score (BMI-SDS) was also calculated using reference data for German children [29].

### Enzyme-linked immunosorbent assay (ELISA)

The following parameters were analyzed in plasma and serum, respectively using a commercially available ELISA kit: leptin, insulin and soluble CD14 (sCD14) (all Hölzel GmbH, Ingolstadt, Germany), D-lactate (Cayman Chemical, Ann Arbor, MI, USA), plasminogen activator inhibitor (PAI)-1 activity (LOXO GmbH, Dossenheim, Germany), tumor necrosis factor *α* (TNF α) (IBL international GmbH, Hamburg, Germany), lipopolysaccharide-binding protein (LBP) (Abnova, Taipei City, Taiwan), adiponectin (TECOmedical AG, Sissach, Switzerland), c-reactive protein (CRP) (DRG Instruments GmbH, Marburg, Germany) and interleukin (IL) -6 (R&D Systems, Abingdon, UK).

### Endotoxin concentrations and small intestinal bacterial overgrowth

Plasma samples were heated for 20 min at 70°C. Endotoxin concentrations were determined by an endpoint enzymatic assay based on limulus amebocyte lysate for a concentration range of 0.015–1.2 EU/ml (LAL) (Charles River, Ecully, France). The mean endotoxin spike recovery rate was 112%. Small intestinal bacterial overgrowth was assessed by performing a glucose H_2_ breath test as described by Volynets *et al*. [13].

### Statistical analysis

Results are presented as mean ± standard error of the mean (SEM) or median (interquartile range [IQR]). Data were analyzed using Mann-Whitney *U* test to compare values between the two groups. Differences in gender, ethnicity and SIBO prevalence between groups were assessed using Fisher´s exact-test. Correlation analysis was assessed using Spearman rank correlation (GraphPad Prism, version 6.02, 2013, GraphPad Software Inc., San Diego, CA, USA). A p-value < 0.05 was considered as significant.

## Results

### Characteristics of study participants

Anthropometric and metabolic characteristics of children enrolled in both groups have been reported in detail already before [27] and are summarized in Table 1. Children in the NAFLD group showed only early signs of hepatic steatosis e.g. grade 1 steatosis. ALT activity being within the normal range was significantly higher in children with early signs of NAFLD than in controls (22 U/l vs. 18 U/l, p< 0.05). In contrast, AST activity was similar between groups (see Table 1). As already reported by Engstler *et al*. before, dietary intake, age, ethnicity and gender did not differ between groups; however, children with NAFLD showed higher height, BMI, BMI-SD score, percentiles and waist circumference (p < 0.05) (Table 1). Moreover, blood levels of triglycerides and insulin as well as HOMA-IR were significantly higher in children showing early signs of NAFLD than in controls. While total cholesterol and LDL cholesterol concentration did not differ between groups, HDL-cholesterol levels were significantly lower in children with early signs of NAFLD than in controls (p<0.05) (see Table 1). However, adiponectin plasma levels did not differ between groups while leptin plasma levels were significantly higher in children with early signs of NAFLD than in controls. Blood pressure was markedly increased in children with NAFLD compared to children without any metabolic abnormalities (see Table 1).

**Table 1:** Characteristics of controls and children with early signs of NAFLD.

	Controls	NAFLD
**n**	29	20
**Sex (male/female)**	15/14	8/12
**Ethnicity (Caucasian/Asian)**	22/7	10/10
**Age (years)**	7.1 (6.6–8.1)	8.25 (6.9–9.0)
**Height (m)**	1.26 (1.21–1.32)	1.32 (1.29–1.39)*
**BMI (kg/m²)**	16.8 (16.2–17.8)	22.1 (19.4–24.2)*
**BMI-SD score**	0.5 (0.27–0.92)	2.14 (1.50–2.38)*
**Percentiles**	72 (60.5–82.5)	98.5 (93.8–99)*
**Waist circumference (cm)**	58.8 (57–61.4)	77.3 (70.2–81.8)*
**Systolic blood pressure (mmHg)**	102.5 (99–109.5)	105 (101–116)
**Diastolic blood pressure (mmHg)**	63 (56.8–66.3)	68 (63.1–73.3)*
**ALT (U/l)**	18 (15–22)	22 (20–28.8)*
**AST (U/l)**	33 (30–37.5)	33 (26.8–35.8)
**Triglycerides (mg/dl)**	53 (43–62.5)	74.5 (64–101)*
**HDL cholesterol (mg/dl)**	56 (52–61.5)	50 (42.5–58.8)*
**LDL cholesterol (mg/dl)**	98 (87–111)	108 (93–123.8)
**Total cholesterol (mg/dl)**	168 (151.5–186.5)	171.5 (157.8–190.3)
**Leptin (ng/ml)**	1.8 (1.1–3)	7.9 (4.4–13.3)*
**Adiponectin (μg/ml)**	10 (6.2–15.7)	9.8 (7.2–13.6)
**Insulin (μIU/ml)**	8.6 (7.1–9.4)	12.1 (10.3–16.6)*
**HOMA-IR**	1.7 (1.6–2)	2.3 (1.9–4)*
**TNF α (pg/ml)**	0.126 (0.104–0.140)	0.141 (0.123–0.172)*
**SIBO (with/without)**^#^	2/27	2/17^#^

BMI: body mass index, BMI-SD score: BMI standard deviation score, ALT: alanine aminotransferase, AST: aspartate aminotransferase, HOMA-IR: homeostatic model assessment for insulin resistance, TNF α: tumor necrosis factor α, SIBO: small intestinal bacterial overgrowth Data are shown as absolute numbers or median (IQR),

*p < 0.05 compared to healthy control children,

^#^one child refused the H_2_ exhalation test.

### SIBO, plasma endotoxin, LBP and D-lactate concentrations as well as serum levels of sCD14

**Fig 1:**
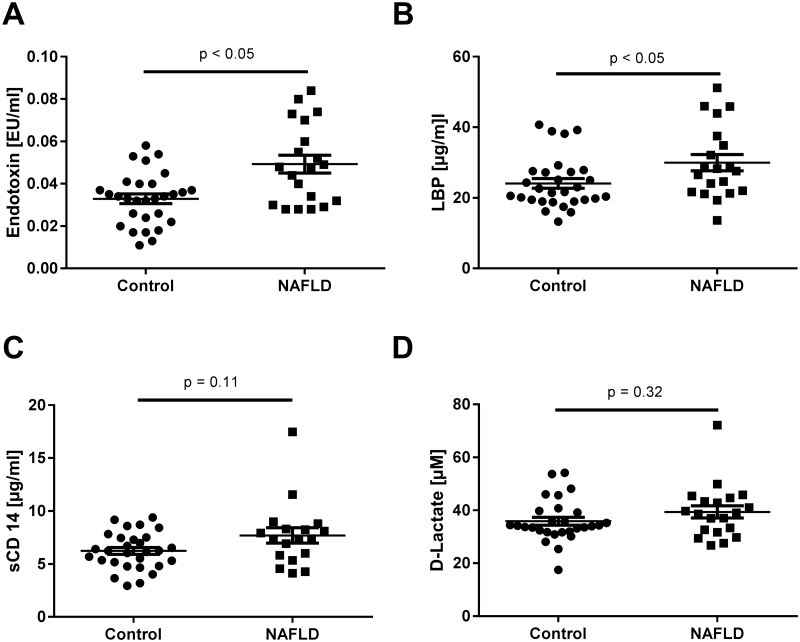
(A) Endotoxin and (B) LBP plasma concentrations as well as (C) sCD14 serum concentrations and (D) D-lactate plasma levels in controls and children with early signs of NAFLD. Data are shown as mean ± SEM, p < 0.05 is considered as significant compared to healthy control children. LBP: lipopolysaccharide-binding protein, sCD14: soluble CD14, NAFLD: non-alcoholic fatty liver disease.

While prevalence of SIBO did not differ between groups, fasting plasma endotoxin concentrations were significantly higher in children suffering from early steatosis than in controls (~+1.5-fold, p < 0.05, see Table 1). Levels of LBP in plasma were also significantly higher in children with early signs of NAFLD than in controls (~+1.2-fold, p < 0.05). In contrast, concentrations of sCD14 in serum and D-lactate levels in plasma did not differ between groups (see Fig 1).

### Concentration of proinflammatory markers in serum and plasma of controls and children with early signs of NAFLD

**Fig 2:**
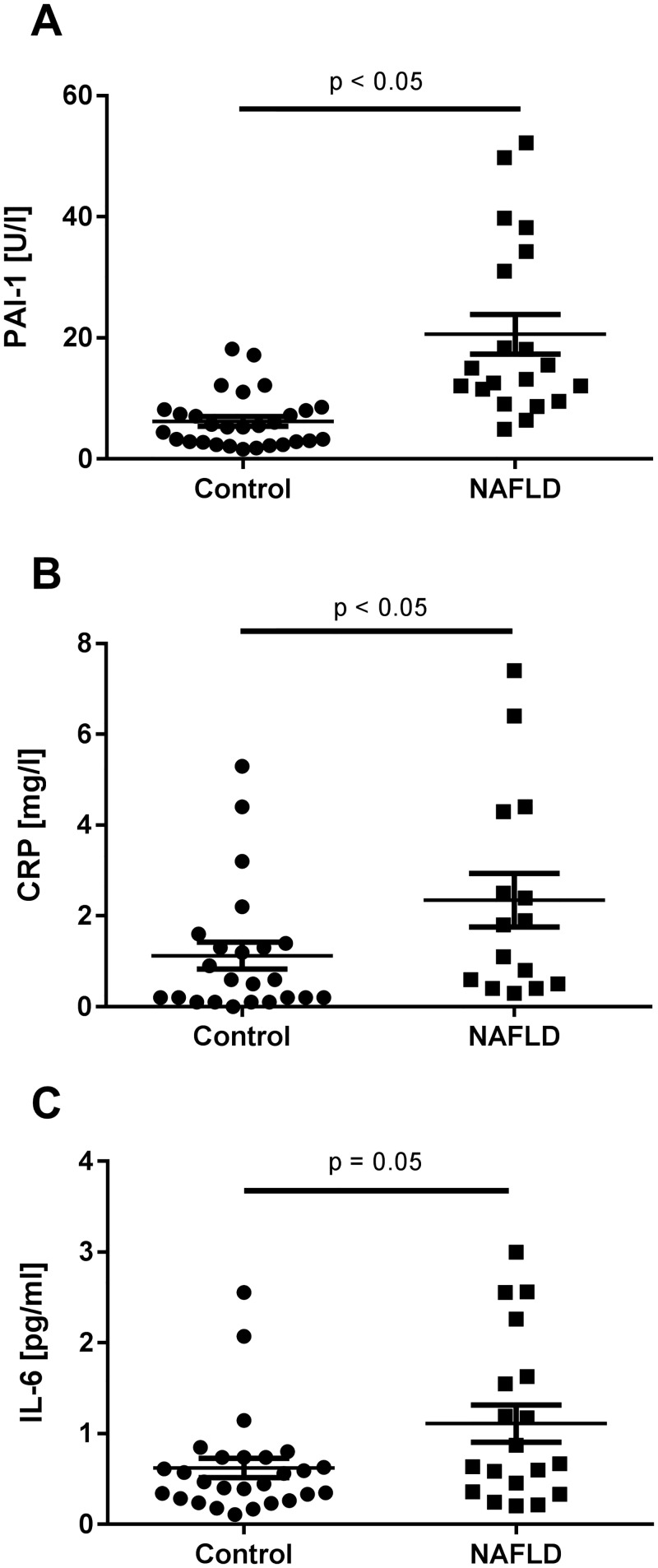
(A) PAI-1 plasma, (B) CRP serum and (C) IL-6 serum concentrations of controls and children with early signs of NAFLD. Data are shown as mean ± SEM, p < 0.05 is considered to be significant compared to healthy control children. For the measurement of IL-6, n = 1 serum sample was insufficient. PAI-1: plasminogen activator inhibitor-1, CRP: c-reactive protein, IL-6: interleukin-6, NAFLD: non-alcoholic fatty liver disease.

Both, PAI-1 activity in plasma (~+3.3-fold, p < 0.05) and CRP serum levels (~+2-fold, p < 0.05) were significantly higher in children with early signs of NAFLD than in controls. Furthermore, in line with the findings for TNF α plasma levels reported before by Engstler *et al*. for this study population, serum IL-6 protein concentrations were by trend higher in children with early signs of NAFLD than in children without any metabolic abnormalities (~+1.6-fold, p = 0.05) (see Fig 2, Table 1) [27].

### Correlation analysis of markers of intestinal barrier function, inflammation and metabolic abnormalities

Results of the correlation analysis are summarized in Table 2. Plasma endotoxin and LBP levels were positively associated with levels of active PAI-1, CRP, IL-6 and leptin plasma and serum levels. LBP plasma levels were positively related to plasma endotoxin and serum sCD14 concentrations. No associations were found for endotoxin and LBP with liver parameters or markers for insulin resistance. Serum levels of sCD14 were positively associated with AST activity, active PAI-1 concentration and markers of insulin resistance (insulin and HOMA) in blood. In contrast, sCD14 levels were negatively related to adiponectin blood concentrations. Soluble CD14 serum levels were not associated with ALT levels, CRP, IL-6 or leptin levels in blood. Furthermore, D-lactate levels were only positively associated with PAI-1, leptin and insulin plasma concentrations.

**Table 2:** Correlation analysis of markers of intestinal barrier function, inflammation and metabolic abnormalities.

	Endotoxin		LBP		sCD14		D-lactate	
	r*	p-value	r*	p-value	r*	p-value	r*	p-value
**Endotoxin**			0.33	**0.02**	0.12	0.43	0.07	0.62
**LBP**	0.33	**0.02**			0.33	**0.03**	0.08	0.58
**sCD14**	0.12	0.43	0.33	**0.03**			0.05	0.71
**ALT**	0.21	0.15	0.19	0.19	0.23	0.13	-0.05	0.72
**AST**	-0.09	0.54	0.16	0.26	0.25	**0.09**	-0.25	0.08
**PAI-1**	0.46	**0.0008**	0.28	**0.05**	0.27	**0.06**	0.40	**0.005**
**CRP**	0.34	**0.04**	0.58	**0.0001**	0.18	0.28	0.29	0.08
**IL-6**	0.28	**0.06**	0.40	**0.005**	0.12	0.44	-0.02	0.90
**Leptin**	0.36	**0.01**	0.26	**0.07**	0.12	0.44	0.34	**0.02**
**Adiponectin**	0.20	0.18	0.04	0.78	-0.33	**0.03**	-0.12	0.40
**Insulin**	0.18	0.21	0.04	0.77	0.35	**0.02**	0.28	**0.05**
**HOMA-IR**	0.16	0.27	-0.02	0.89	0.27	**0.07**	0.20	0.19

LBP: lipopolysaccharide-binding protein, sCD14: soluble CD14, ALT: alanine aminotransferase, AST: aspartate aminotransferase, PAI-1: plasminogen activator inhibitor-1, CRP: c-reactive protein, IL-6: interleukin-6, HOMA-IR: homeostatic model assessment for insulin resistance

*Spearman R.

## Discussion

Results of several human studies suggest that later stages of the disease e.g. manifest steatosis and steatohepatitis with and without beginning fibrosis are frequently associated with increased bacterial endotoxin levels in peripheral blood suggesting that intestinal barrier dysfunction may be critical in patients with NAFLD [8, 12, 13]. However, if these alterations are causally involved in disease initiation or develop with time has not yet been fully understood. In the present study, we were able to show that even in juvenile patients with beginning NAFLD e.g. in patients with simple steatosis grade 1 and no marked increase of ALT and AST, plasma endotoxin and LBP plasma levels are significantly higher than in controls. Furthermore, correlation analysis revealed that levels of LBP and endotoxin were strongly correlated. Importantly, as we only enrolled children below the age of 10 years, lifestyle factors shown to affect the development of NAFLD like drug intake, smoking and alcohol ingestion [22, 23] were not yet present. However, despite only showing very early signs of NAFLD and in line with findings of others [8, 9], prevalence of fatty liver stage 1 in children was associated with higher blood pressure, beginning insulin resistance and dyslipidemia as well as increased levels of active PAI-1, CRP, IL-6, TNF α and leptin. Neither sCD14 nor D-lactate levels differed significantly between groups. The latter parameter has been discussed to be indicative of intestinal permeability in patients with liver cirrhosis and alcoholic liver disease [32, 33]. However, to date, systematic studies determine D-lactate levels in patients with NAFLD are lacking. Therefore, it cannot be ruled out that measurements of D-lactate are more suitable to determine severe alterations of intestinal barrier function probably not yet present in subjects enrolled in the present study. Our findings for sCD14 are in line with those of others, also showing that despite significantly higher endotoxin levels, sCD14 levels were not altered in patients with early phases of the disease e.g. steatosis when compared to healthy controls [34]. Still sCD14 serum levels and LBP plasma concentration were positively related, while in line with others [35], no association of sCD14 and endotoxin plasma levels was found. Also in line with the present study, de Courten *et al*. reported a positive association of sCD14 and LBP [35]. Furthermore, prevalence of SIBO was similar between groups suggesting that in the present study SIBO might not have been a key mediator of the increased bacterial endotoxin levels found in young children with early signs of NAFLD. However, it cannot be ruled out that in other settings and later stages of the disease, SIBO might contribute to changes in the gut barrier function. Also, assessment of SIBO was only performed using a glucose breath test, which only allows for a rough estimation of the presence of SIBO [36]. Indeed, others have shown before in adults and children as well as adolescents, that SIBO in patients with NAFLD determined with methods like the one used in the present study is not always associated with an increased permeability and/ or elevated endotoxin levels in plasma [10, 13]. Taken together, our results suggest that an increased translocation of bacterial endotoxin is not only found in patients with more severe stages of NAFLD but might actually play an initial role in the onset of NAFLD.

### Markers of intestinal barrier function are related to metabolic and inflammatory markers

Elevated endotoxin levels have been shown before to be associated with increased levels of active PAI-1 and induced PAI-1 mRNA expression in liver tissue [12, 37]. In the present study, LBP, sCD14 and D-lactate levels were all positively related to active PAI-1 levels. In support of these findings, results of animal studies suggest that LBP and CD14 are critical in the endotoxin-dependent induction of PAI-1, while for D-lactate, similar findings are lacking [38, 39]. Furthermore, LBP and endotoxin plasma levels were positively related to CRP and IL-6 serum levels. Positive associations of CRP, IL-6 and LBP have been reported before by others [40–42]; however, in these studies, patients suffered from severe liver disease e.g. cirrhosis with infection. In contrast to the findings for endotoxin and LBP but in line with the findings of Harte *et al*. in adults with biopsy proven NAFLD, levels of sCD14 were positively related to markers of insulin resistance whereas adiponectin levels were negatively related to sCD14 levels [34]. Indeed, CD14 has repeatedly been suggested before to be associated with the prevalence of obesity and insulin resistance [43, 44]. Taken together, results of our study suggest that endotoxin and LBP plasma levels are strongly related to proinflammatory changes even in the early stages of NAFLD, while sCD14 levels seem to be associated with markers of insulin resistance. Molecular mechanisms involved in the correlations found in the present study remain to be determined.

### Limitations

Our study is not without limitations. Indeed, a major limitation is the rather small study population including only 20 children with early stages of NAFLD and 29 control children, who were primarily Caucasian. Therefore, it cannot be ruled out that in larger populations with mixed or other ethnic background results might differ. However, despite the small sample size our findings are in line with others showing an association of alterations of intestinal or fecal microbiota composition and intestinal barrier dysfunction in more progressed stages of the disease e.g. steatosis grade 3 or even steatohepatitis with and without fibrosis in children and adolescents as well as adults [8, 10, 12, 13, 45]. The second major limitation is that NAFLD was not biopsy-proven but rather diagnosed by hepatic ultrasound and the elevation of serum transaminase activity. However, for ethical reasons it was not possible to obtain liver biopsies from children especially at the young age of children enrolled in the present study. Indeed, due to its non-invasiveness and safety as well as its accuracy and reliability to detect fatty liver disease, ultrasonography was the technique of choice [46]. Therefore, it cannot be ruled out that in some children inflammatory changes in the liver might have been missed. Furthermore, for ethical and compliance reasons only indirect markers of gut permeability e.g. plasma endotoxin and D-lactate levels were used to determine intestinal barrier dysfunction. In the present study, assessment of SIBO was only performed using a glucose breath test for H_2_ exhalation. As discussed in detail by Saad *et al*. this kind of SIBO test only allows for a rough estimation of the presence of SIBO in the small intestine [36]; however, using other markers would have implied an additional fasting and study day for children and guardians, which was not possible due to a lack of compliance. A further limitation might be the fact that food intake, shown previously to be similar between groups [27], was only assessed by two independently preformed 24 h recalls. Indeed, it has been shown that 24 h recalls might miss foods consumed irregularly [47] which still might impact intestinal barrier function or microbiota; however, in a pilot study (unpublished data), we found that 24 h recalls are the most suitable measure to assess dietary intake in children below the age of 10 without overly straining children and guardians.

## Conclusion

Taken together, results of the present study further bolster the hypothesis that alterations of intestinal barrier function are critical in the development of NAFLD. However, our results also suggest that these alterations are already found in the very early stage of the disease e.g. even in the absence of any liver related abnormalities like increased transaminases or significant hepatic fat accumulation. Our findings also suggest that targeting changes of intestinal microbiota, be it through the use of probiotics or altered dietary pattern like increasing the intake of fiber might be beneficial in the prevention and therapy of NAFLD in children. However, molecular mechanisms causing intestinal barrier dysfunction remain to be determined.
